# A retrospective study on parapneumonic effusion in 130 dogs with a clinical diagnosis of pneumonia

**DOI:** 10.3389/fvets.2023.1144148

**Published:** 2023-03-16

**Authors:** Priscilla Burnotte, Nicolas Graziano, Kris Gommeren

**Affiliations:** Department of Small Animal Clinical Science, Faculty of Veterinary Medicine, University of Liège, Liège, Belgium

**Keywords:** parapneumonic effusion, pneumonia, dogs, C-reactive protein (CRP), outcome

## Abstract

**Objective:**

To screen the occurrence of parapneumonic effusion in dogs.

**Methods:**

Medical records were searched for dogs with a presumptive diagnosis of bacterial pneumonia from 2017 to 2021 at the Liege university teaching hospital. Bacterial pneumonia was presumptively diagnosed based on compatible clinical signs and findings; thoracic radiographs compatible with bacterial bronchopneumonia; and either increased serum C-reactive protein (CRP) levels, a positive bronchoalveolar lavage culture or a positive clinical evolution in response to antibiotic therapy. Patients diagnosed with parasitic or other non-bacterial inflammatory pneumonia or with pulmonary neoplasia were excluded. Signalment, clinical findings, and outcome were recorded.

**Results:**

One hundred and thirty dogs were included in the study, of which 44 dogs (33.8%) developed a parapneumonic effusion. Four of these dogs (4/44; 9%) had thoracocentesis performed, displaying a modified transudate (2) or septic exudate (2).

**Conclusions:**

Although parapneumonic effusion in dogs with a presumptive diagnosis of bacterial pneumonia appears to be rather common (33.8%), thoracocentesis or chest tube placement was rarely performed. Furthermore, the outcome of dogs with and without parapneumonic effusion appears to be similar.

## 1. Introduction

Bacterial pneumonia is defined as inflammation of the pulmonary parenchyma secondary to a bacterial infection (1). Classification of pneumonia usually is based on anatomic location (lung parenchyma or bronchi), or underlying mechanisms associated with infection: aspiration pneumonia (AP), community-acquired pneumonia (CAP), hematogenous spread of bacteria, foreign body, or immunodeficiency. Reportedly, the two most common types of bacterial pneumonia in dogs are CAP and AP (2).

Parapneumonic effusion is defined as the accumulation of free fluid in the pleural space in association with a simultaneous diagnosis of bacterial pneumonia. The incidence of parapneumonic effusion of varying severity in human patients is reported to be up to 40–60%. Parapneumonic effusions are classified into three categories (3). Uncomplicated parapneumonic effusions, which are exudative, neutrophilic effusions, associated with a negative cytology and culture; complicated parapneumonic effusions, resulting from a small amount of bacteria; Empyema thoracis in which there is frank pus in the pleural space with positive cytology and culture (3). Prompt antibiotic therapy may prevent the development of parapneumonic effusions as well as the progression of (un)complicated parapneumonic effusions into Empyema thoracis.

In veterinary medicine, parapneumonic effusion associated with bacterial pneumonia in dogs is rarely described in scientific publications. Information regarding the incidence, management the correlation of the identification of parapneumonic effusion and acute phase protein levels, as well as the impact of parapneumonic effusion on patient outcome is lacking.

The primary aim of this retrospective study was to assess the incidence of parapneumonic effusion in dogs with a clinical diagnosis of pneumonia in a veterinary university clinic. The second objective was to report whether these patients required thoracocentesis and thoracic drain placement. A third objective was to compare serum C-reactive protein (CRP) concentrations in dogs with and without parapneumonic effusion. A final objective was to compare the outcome of dogs with and without parapneumonic effusion.

## 2. Methods

A retrospective review of medical records of dogs with a presumptive diagnosis of bacterial pneumonia over a 4-year period (January 2017–December 2021) presented to the Veterinary Small Animal Teaching Hospitals of the Faculty of Liège (Belgium) was conducted. Collected data included signalment, physical examination findings, serum CRP concentrations, thoracic X-ray reports, bronchoalveolar lavage fluid (BALF) analysis and thoracic point of care ultrasound (Thoracic POCUS) reports if available. All thoracic X-rays were assessed by residents and final reports validated by a board-certified radiologist. The amount of pleural effusion on thoracic X-rays was subjectively assessed, based on the following criteria: a small amount of pleural effusion was defined by the visualization of one or more thin interlobar pleural fissures and/or a small degree of lung retraction from the chest wall and/or focal border effacement by a fluid opacity band of the cardiac and diaphragmatic silhouettes. Moderate pleural effusion was defined by the identification of wider pleural fissures and/or a greater degree of lung retraction from the chest wall and/or wider border effacement of the cardiac and diaphragmatic silhouettes. Severe pleural effusion was defined by the visualization of thick pleural fissures and/or a very high degree of retraction of the lungs from the chest wall and/or a major border effacement of the cardiac and diaphragmatic silhouettes.

A presumptive diagnosis of bacterial pneumonia was based on compatible clinical signs (e.g., cough, fever, increased respiratory rate, and acute dyspnoea) and clinical findings; thoracic radiographs compatible with bacterial bronchopneumonia; and increased serum CRP levels, a positive bronchoalveolar lavage culture or a positive clinical evolution in response to antibiotic therapy. Serum samples obtained for CRP analysis were immediately analyzed (IDEXX catalyst CRP). The upper reference range was <10 mg/L. Dogs with a final diagnosis of inflammatory non-septic pneumonia, parasitic pneumonia, or pulmonary neoplastic disease were excluded from the study. The record of each patient was reviewed for the description of pleural effusion on the report of thoracic X-rays and Thoracic POCUS.

Data analysis was conducted using a commercial software program (XLStat, ADDINSOFT). Data were expressed as median and range (non-normally distributed) or mean and standard deviation (SD, normally distributed). For categorical data, groups were compared using the Chi squared (χ^2^) test. A *p*-value < 0.05 was considered significant.

## 3. Results

A total of 220 records were reviewed for the purpose of this study. Ninety dogs were excluded, 65 had a final diagnosis of an inflammatory non-infectious pneumonia, 15 dogs had parasitic pneumonia, and 10 had neoplastic disease. One hundred and thirty dogs of various breeds met the inclusion criteria. French Bulldog (14/130; 10.7%), English Bulldog (12/130; 9.2%), Great Dane (9/130; 6.9%), Golden Retriever (6/130; 4.6%), and Cavalier King Charles Spaniel (5/130; 3.8%), as well as mixed breeds (11/130; 8.5%) were the most common breeds. The median age of dogs was 5.7 years (range 1 month−15 years).

Commonly observed clinical signs are displayed in Table 1. The most common reported clinical signs at presentation were unspecific findings such as lethargy (126/130; 96.9%) and anorexia (122/130; 93.8%) in the majority of dogs included. More specific findings such as hyperthermia (80/130; 61.5%), and respiratory signs such as tachypnea (92/130; 70.1%), cough (43/130; 33.1%), and mild to severe dyspnea (32/130; 24.6%) were still observed in many patients, often in a combination of these findings. The final diagnosis of included patients was AP in 107 dogs (107/130; 82.3%), CAP in 15 dogs (15/130; 11.5%) and bronchopneumonia (BP) as a complication of a chronic primary pulmonary disease in 8 dogs (8/130; 6.2%). All dogs with a final diagnosis of aspiration pneumonia had a history of acute vomiting (74/107; 69.2%) or a predisposing condition or event such as a megaoesophagus (13/107; 12.1%), brachycephalic airway obstructive syndrome associated with chronic regurgitation (11/107; 10.3%), anesthesia (5/107; 4.7%), or laryngeal paralysis (4/107; 3.7%).

**Table 1:** Main clinical signs observed in dogs with bacterial pneumonia at presentation.

**Clinical signs**	**Number of dogs (*n* = 130)**	**Percentiles (%)**
Lethargy	126	97
Anorexia	122	94
Tachypnea	92	71
Hyperthermia	80	61
Cough	43	33
Dyspnea	32	25

Reports of thoracic X-rays were available for all included dogs. In dogs with AP a cranioventral alveolar pattern was described, either localized on the right (57/107; 53.2%) or left (15/107; 14.0%) side or bilateral in (35/107; 32.7%), with the right median lobe being the most commonly affected (61/107; 57.0%). In dogs with CAP a bilateral caudodorsal diffuse interstitial (10/15; 66.7%) or a cranioventral alveolar (5/15; 33.3%) pattern was described. Finally in dogs with BP there was no typical pattern described.

BALF was performed on 19 dogs (19/130; 14.6%), with a positive bacterial culture obtained in 15 dogs (15/19; 78.9%). CRP concentrations were available for 118 patients (118/130; 90.8%), with concentrations ranging from 25.2 to 532 mg/L.

**Figure 1:**
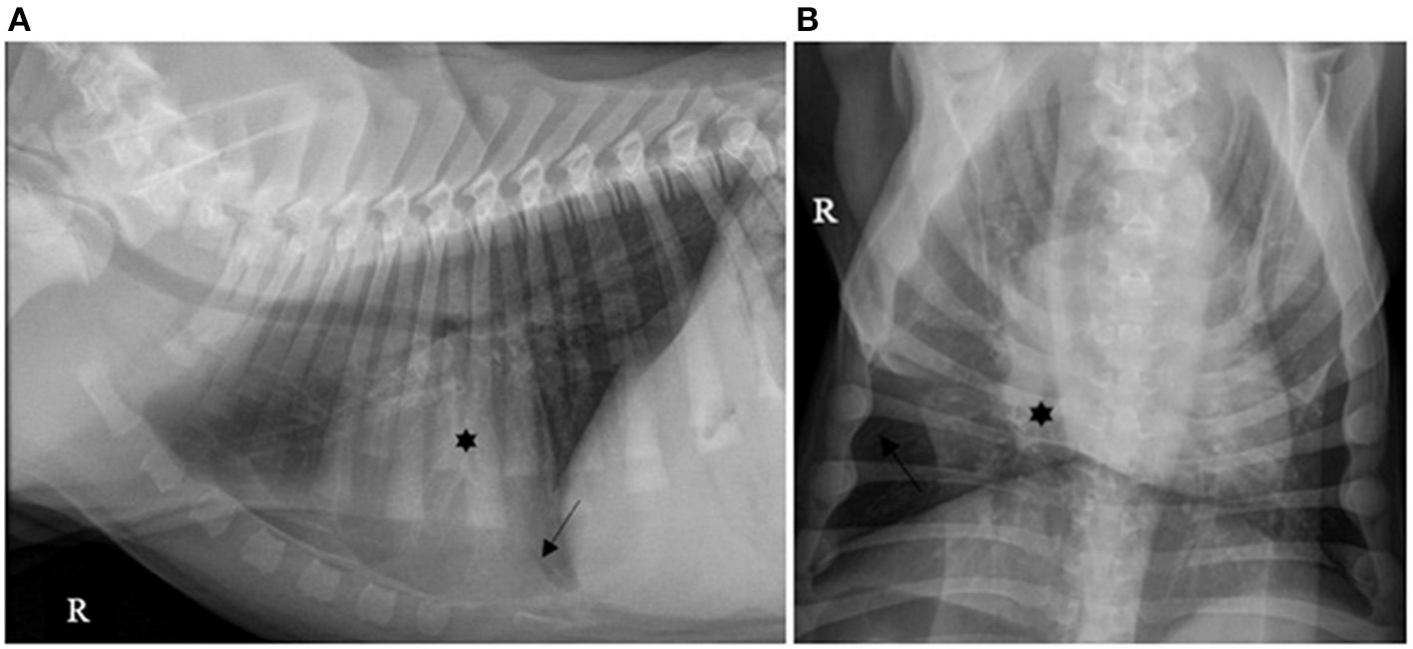
**(A)** Right lateral and **(B)** ventro-dorsal thoracic X-rays of a dog with AP associated with a small amount of PE. A discrete alveolar pattern is visible in the right middle lobe (*). A discrete PE is identified by identification of thin fissure lines between the lobes (dark arrows). AP, aspiration pneumonia; PE, pleural effusion (source: medical imaging department of the veterinary university clinic of Liege, Belgium).

**Figure 2:**
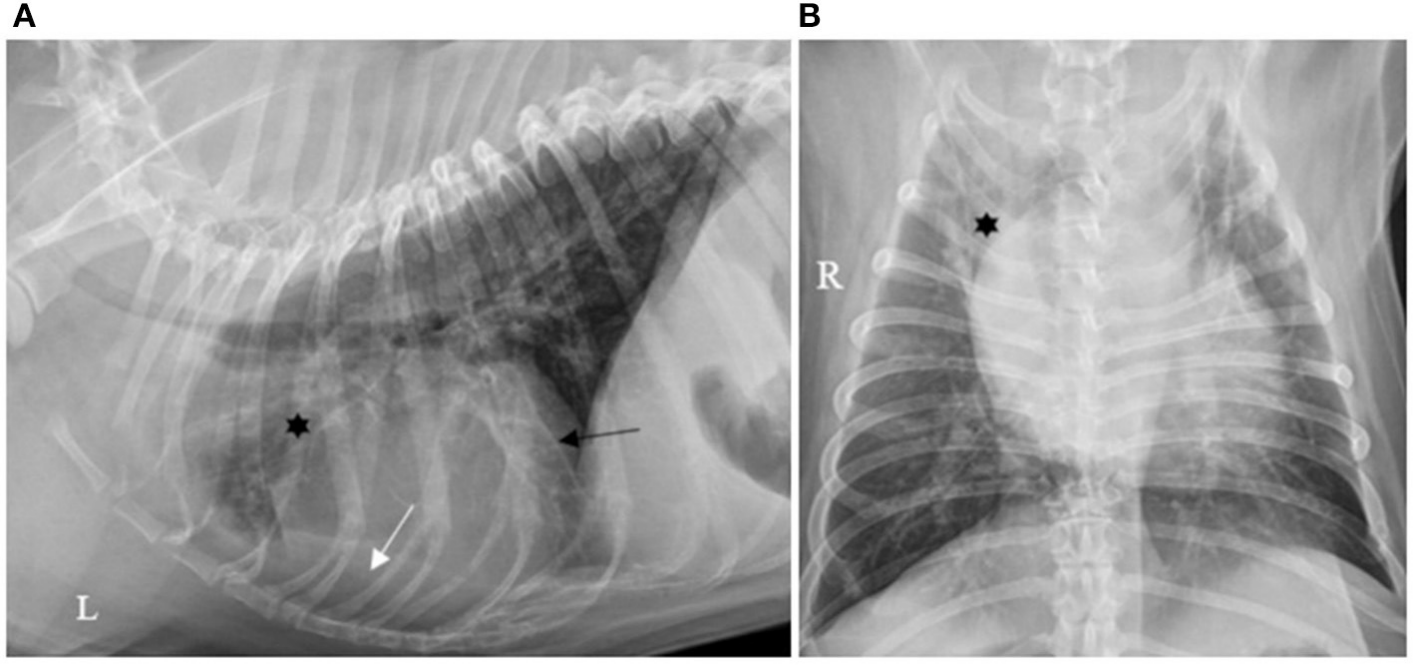
**(A)** Left lateral and **(B)** ventro-dorsal thoracic X-rays of a dog with AP associated with a moderate amount of PE. An alveolar pattern is distributed ventrally in the right cranial lobe (*). A moderate amount of PE is identified by identification of a thicker fissure line (dark arrow) associated with border effacement by a fluid opacity band of the cardiac silhouette ventrally (white arrow). AP, aspiration pneumonia; PE, pleural effusion (source: medical imaging department of the veterinary university clinic of Liege, Belgium).

**Figure 3:**
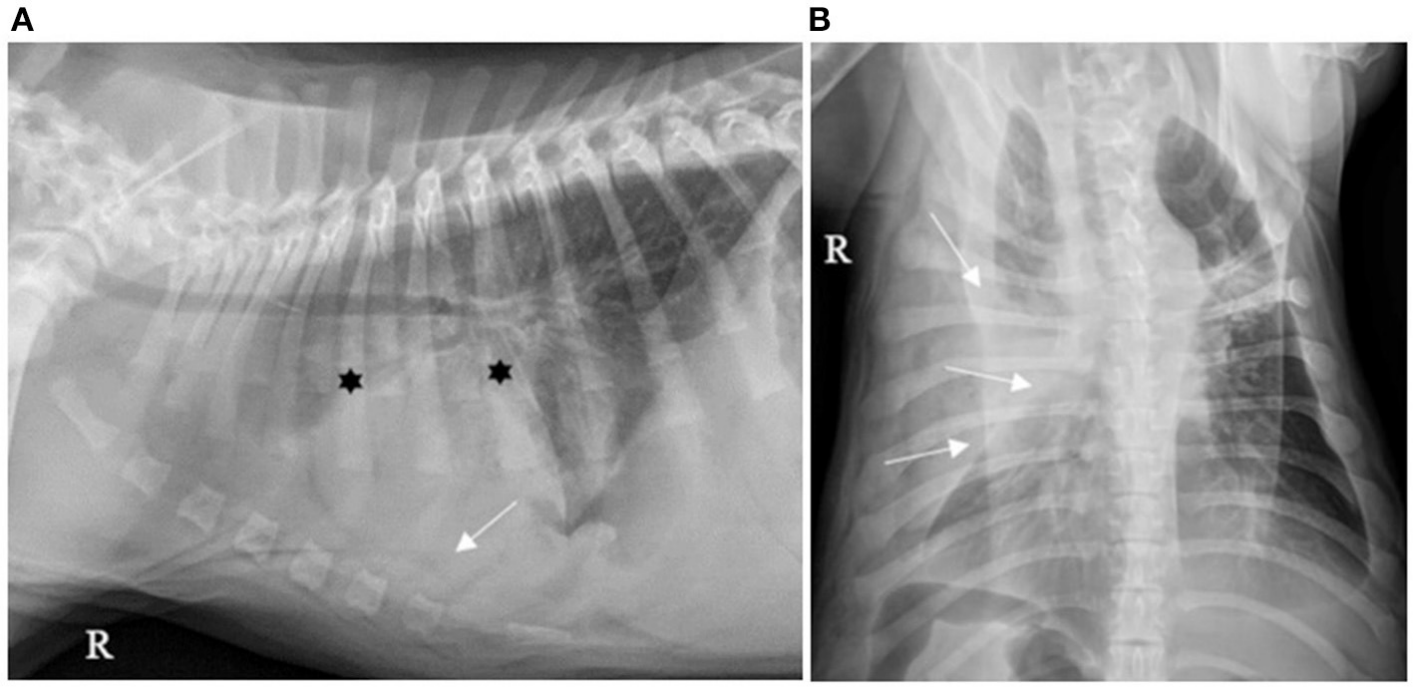
**(A)** Right lateral and **(B)** ventro-dorsal thoracic X-rays of a dog with AP associated with severe PE. An alveolar pattern is mainly distributed cranioventrally in the right cranial and middle lobes (*). PE is identified by thick pleural fissure lines associated with border effacement by a fluid opacity band, ventral to the cardiac and diaphragmatic silhouettes and lateral to the right chest wall, pushing the lung lobes medially (white arrows). AP, aspiration pneumonia; PE, pleural effusion (source: medical imaging department of the veterinary university clinic of Liege, Belgium).

Forty (40/130; 30.7%) cases had pleural effusion identified on the reports of their thoracic X-rays, which was described as a small amount in 36/40 (90.0%), moderate in 2/40 (5.0%) and severe in 2/40 (5.0%), as illustrated by Figures 1–3.

**Figure 4:**
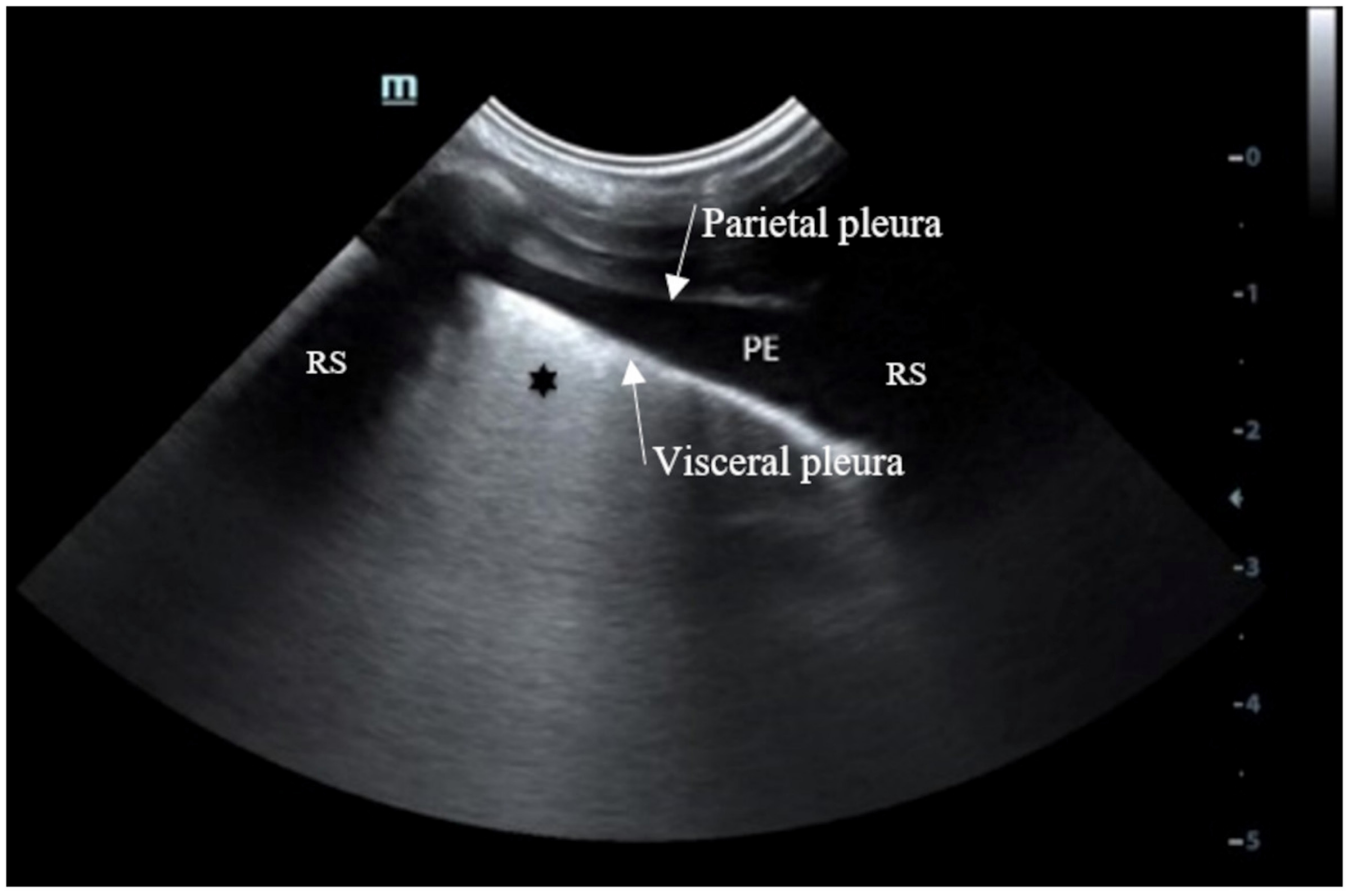
Thoracic POCUS image with the probe oriented perpendicular between two ribs, located in the lowest part of the thorax of a dog with a presumptive diagnosis of AP with PE. Anechoic free fluid (PE) is identified between the parietal and visceral pleura. The visceral pleura appears thicker with coalescing B lines (*). AP, aspiration pneumonia; PE, pleural effusion; RS, Rib shadow (source: emergency and critical department of the veterinary university clinic of Liege, Belgium).

Thoracic POCUS was performed in 91/130 (70.0%) dogs, either as described by Fernandes Rodrigues et al. (4) or according to the PLUS technique (5). Pleural effusion was described in 29/91 (31.8%) that had thoracic POCUS performed. An example of thoracic POCUS image of a dog consistent with AP associated with parapneumonic effusion is shown in Figure 4.

Parapneumonic effusion was detected in 44/130 (33.8%) of dogs, with 25 (25/44; 56.8%) dogs having pleural effusion reported on thoracic X-rays and thoracic POCUS, 4 (4/44; 9%) only on thoracic POCUS, and 15 (15/44; 34.1%) only on thoracic X-rays.

Despite the high incidence of pleural effusion, thoracocentesis (4/44; 9%), and chest tube placement (2/44; 4.5%) was rarely performed. Thoracocentesis was reportedly performed in order to analyze the fluid in all 4 cases, and to evacuate the fluid and improve breathing in 2 of these cases, which subsequently had a thoracic drain placed.

**Figure 5:**
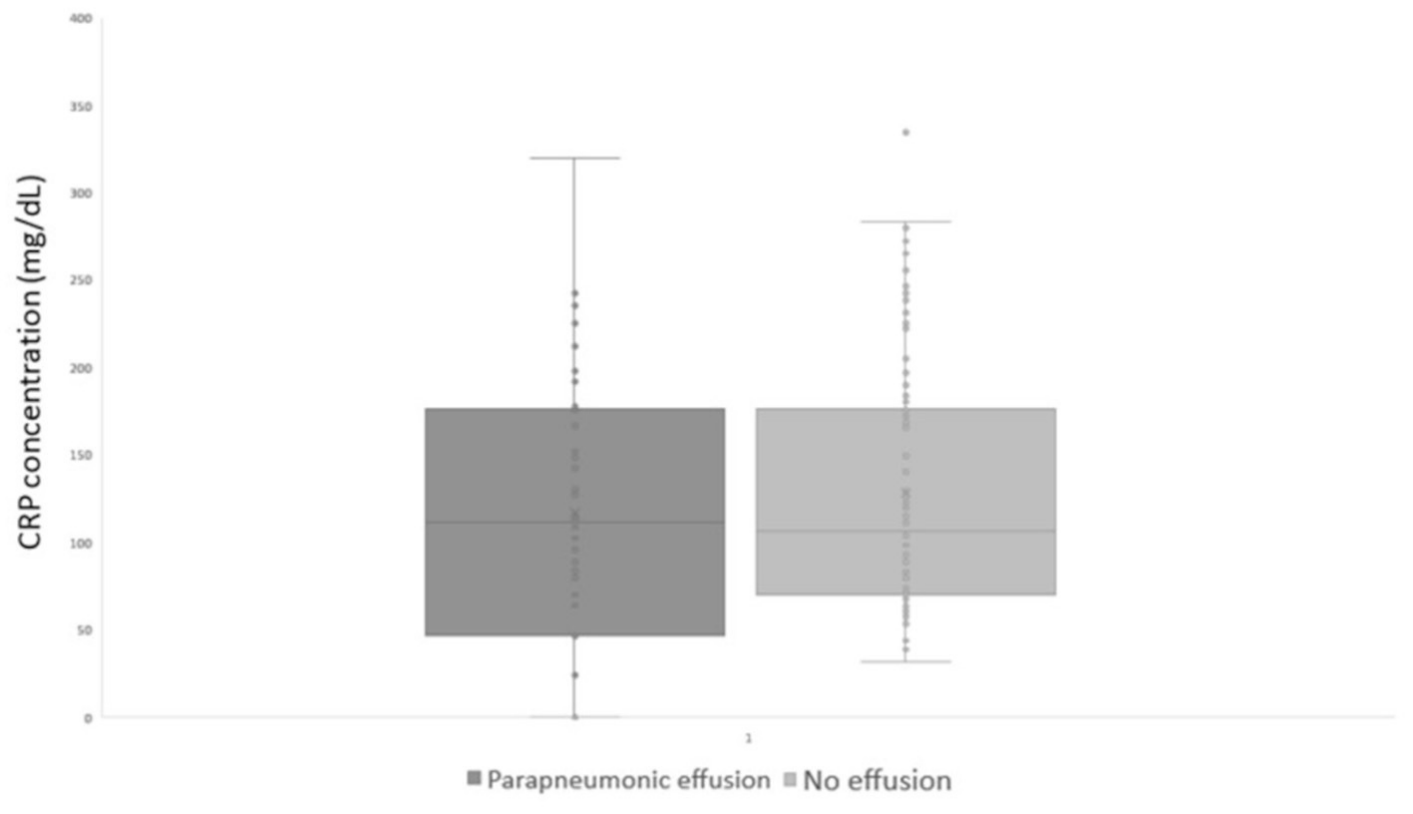
CRP at diagnosis in dogs with and without parapneumonic effusion. CRP, Serum C-Reactive protein.

The median (range) CRP level of dogs with and without parapneumonic effusion was not significantly different [127.6 mg/L (25.2–532) and 110.9 mg/L (31.5–462); *p* = 0.35; Figure 5]. Overall mortality of dogs with a clinical diagnosis of pneumonia was 29/130 (22.3%), all of which were diagnosed with AP. Mortality rates in dogs with and without parapneumonic effusion were not significantly different (*p*-value 0.56), at 8/44 (18.1%) and 21/86 (24.4%), respectively.

## 4. Discussion

To the authors' knowledge, this is the first study to retrospectively describe the incidence of parapneumonic effusion in dogs with a presumptive diagnosis of bacterial pneumonia.

Brachycephalic dogs (including French and English bulldogs), and giant breed dogs represented 27% of our population. This might be explained by the high proportion of dogs included with an AP compared to CAP, as brachycephalic dogs and giant breed dogs both are predisposed to AP (6, 7). According to Darcy et al. brachycephalic breeds often suffer from brachycephalic obstructive airway syndrome, possibly in combination with secondary gastro-esophageal abnormalities, which both have been identified as risk factors for the development of AP (6). Among giant breed dogs, the Irish Wolfhound has been shown to have the highest breed incidence of aspiration pneumonia. Greenwell et al. recommend that if no obvious underlying disease process is identified, subclinical laryngeal paralysis should be considered (7).

The distribution of thoracic X-rays lesions in our population was typical for AP or CAP for the majority of cases. However, four dogs with a cranioventral alveolar pattern had a final diagnosis of CAP, based on a BALF culture positive for *Bordetella bronchiseptica*.

Parapneumonic effusion was diagnosed in 44 out of 130 patients (33.8%) in this single center retrospective study. Previous veterinary publications on bacterial pneumonia did not report on the presence of pleural effusion in dogs (4, 8, 9). It is unclear whether this was due to a true lack of parapneumonic effusion, or whether this was not assessed in these cases.

The development of parapneumonic effusion in human medicine is hypothesized to occur secondary to pleural inflammation, leading to neutrophil accumulation and release of proinflammatory cytokines (IL-8, TNF-a). This may subsequently induce endothelial lesions and increased capillary permeability (10, 11). In this retrospective study, inflammatory cytokine concentrations were not measured in any of our patients.

The CRP concentrations of dogs with and without parapneumonic effusion in our study were not statistically different (*p* = 0.35). In human medicine, CRP concentrations have been correlated with the severity of pneumonia (12), but there are no publications demonstrating a correlation with the presence or type of parapneumonic effusion. CRP is an acute phase response, that rises in response to rising concentrations of pro-inflammatory cytokines, especially IL-6. Based on our results, the presence or absence of parapneumonic effusion therefore does not appear to be directly attributable to the degree of inflammation. Indeed, we do believe that other factors such as the type and load of bacteria, the distribution of the infection, as well as patient characteristics such as the breed, immune response and others may all play a role in the development of parapneumonic effusion.

Most patients (118/130; 90.7%) had a presumptive diagnosis based on compatible clinical signs and findings, compatible X-ray findings, and increased CRP concentrations. Nineteen dogs (19/130; 14.6%) had BALF analysis performed, resulting in a positive bacterial culture in 15 dogs (15/19; 78.9%). All 4 patients with a negative culture were under antibiotic therapy at the time of BAL procedure and demonstrated a good clinical evolution under antibiotic therapy, similarly to the 3 patients that did not have CRP or BALF analyzed.

Pleural effusion was detected in 40/130 (30.7%) patients on thoracic X-rays, and 29/130 (22.3%) on thoracic POCUS. Twenty-five patients (25/44; 56.8%) had pleural effusion on both, fifteen (15/44; 34.1%) only on thoracic X-rays and 4/44 (9%) only on thoracic POCUS. In this retrospective clinical study, none of the patients had a computed tomography (CT) performed, considered the gold standard to assess for the presence of even small amount of pleural effusion. The true incidence of pleural effusion therefore likely is higher than the incidence reported on radiography and ultrasound reports.

The majority of parapneumonic effusions in this study were reported on thoracic X-rays (40/44; 90.9%). Thoracic radiography has been reported to have a poor sensitivity (43%) to detect pleural effusion in veterinary medicine (3). In human medicine a meta-analysis of Yousefifard et al. similarly reported a low sensitivity (51%) and high specificity (91%) for thoracic radiographs to detect pleural effusion, in a heterogenous population of patients presenting for trauma, cardiac and intensive care unit (ICU) patients (13). Therefore, in our study the true incidence of parapneumonic effusion was likely higher than the proportion detected on thoracic X-rays.

Thoracic POCUS reported pleural effusion in 29/91 (31.8%) dogs. In veterinary medicine, the sensitivity of thoracic POCUS to detect pleural effusion has been described to be 66.6% (2), whilst a different publication described a fair to moderate agreement between thoracic POCUS and CT for the detection of pleural effusion (14). However, these studies reported pleural effusion in trauma patients.

Due to the retrospective nature, we cannot exclude that pleural effusion was observed, but not reported as considered irrelevant. Thoracic POCUS is a clinical driven technique and does not aim to replace a complete and comprehensive ultrasound. Subsequently, clinicians performing thoracic POCUS on a dyspneic patient suspecting an AP look for cranioventral shred signs and B lines, not necessary for pleural effusion. Scant amounts of pleural effusion may therefore have been missed, or not reported as considered irrelevant for patient management. Secondly, several protocols have been used to perform thoracic POCUS. The technique described by Fernandes Rodrigues et al. was specifically designed to study dogs with aspiration pneumonia and compare thoracic POCUS findings with thoracic X-rays (4). The PLUS protocol describes a technique that is guided by the landmarks of the thoracic cavity and aims to allow for identification of small amounts of free air or fluid (5). Unfortunately, we were unable to assess which patients underwent what type of thoracic POCUS protocol and cannot discuss the performance of each technique to diagnose pleural effusion. Thoracic POCUS is performed by the consulting veterinarian (e.g., an emergency and critical care clinician, resident, or a rotating intern), not by an imager. The experience of the clinician performing thoracic POCUS may have influenced our findings.

The incidence of parapneumonic effusion in human medicine is affected by other factors such as the administration of like non-steroidal anti-inflammatory (15), but this was not assessed in this retrospective paper. Regardless, despite the rather high incidence of parapneumonic effusion in our study, thoracocentesis 2/130 (1.5%) or thoracic chest tube placement 2/130 (1.5%) was rarely performed.

Overall mortality was 22.3% (29/130) with no significant difference between dogs with or without parapneumonic effusion. The reason for death was reported as cardiac arrest in 10/29 (34.4%) and euthanasia because of a guarded prognosis 17/29 (58.6%) or due to financial reasons in 2/29 (6.8%) dogs. All fatal outcomes occurred in the AP group. As mortality rates for dogs with AP and CAP have been reported to vary from 6 to 25% (CAP) and around 12% CAP). Although the outcome of patients was in line with earlier reports (9, 16, 17), the difference between dogs with AP and CAP seems important. The retrospective nature of this study did not allow us to identify possible explanations for this difference.

Compared to other publications, the proportion of dogs that underwent BALF was rather low. BALF would allow to identify the infectious pathogen, and guide antibiotic therapy according to culture and sensitivity tests, which could have a positive effect on outcome.

This retrospective study has several limitations. The incidence of parapneumonic effusion was assessed on X-ray and thoracic POCUS reports as CT scans (considered the gold standard) were not performed. Thoracic X-rays were not reviewed, as original reports were validated by a board-certified radiologist. Thoracic POCUS was performed by the consulting veterinarian, and cineloops were not available for review. The sensitivity of thoracic POCUS to detect pleural effusion in conditions other than trauma has not been reported in dogs. In human medicine two meta-analyses both reported a high sensitivity (94 and 91%) and specificity (98 and 92%) to detect pleural effusion with thoracic POCUS, in trauma, cardiac disease and ICU patients as well as in ventilated patients with primary respiratory disease (13, 18).

## 5. Conclusion

In conclusion, in this single center retrospective observational study, the incidence of parapneumonic effusion in dogs with a presumptive diagnosis of bacterial pneumonia was 33.8%. Although parapneumonic effusion was common, thoracocentesis and fluid analysis was rarely performed. Moreover, CRP levels and outcome of dogs with or without parapneumonic effusion were not significantly different.

## Data availability statement

The original contributions presented in the study are included in the article/supplementary material, further inquiries can be directed to the corresponding author.

## Ethics statement

Ethical review and approval was not required for the animal study because only medical records were reviewed retrospectively.

## Author contributions

PB, KG, and NG contributed to conception and design of the study. PB and NG organized the database and performed the statistical analysis. NG wrote the first draft of the manuscript. PB and KG contributed to manuscript revision. All authors contributed to the article and approved the submitted version.
